# Development of a vendor neutral MRI distortion quality assurance workflow

**DOI:** 10.1002/acm2.13735

**Published:** 2022-07-26

**Authors:** Amy Walker, Phillip Chlap, Trent Causer, Faisal Mahmood, Jarryd Buckley, Lois Holloway

**Affiliations:** ^1^ Liverpool and Macarthur Cancer Therapy Centres Sydney Australia; ^2^ Ingham Institute of Applied Medical Research Sydney Australia; ^3^ Centre for Medical Radiation Physics University of Wollongong Wollongong Australia; ^4^ South Western Clinical School University of New South Wales Sydney Australia; ^5^ Illawarra Cancer Care Centre Wollongong Australia; ^6^ Laboratory of Radiation Physics, Department of Oncology Odense University Hospital Odense Denmark; ^7^ Department of Clinical Research University of Southern Denmark Odense Denmark; ^8^ Institute of Medical Physics University of Sydney Sydney Australia

**Keywords:** distortion, MRI, phantoms, quality assurance

## Abstract

With the utilization of magnetic resonance (MR) imaging in radiotherapy increasing, routine quality assurance (QA) of these systems is necessary. The assessment of geometric distortion in images used for radiotherapy treatment planning needs to be quantified and monitored over time. This work presents an adaptable methodology for performing routine QA for systematic MRI geometric distortion. A software tool and compatible protocol (designed to work with any CT and MR compatible phantom on any scanner) were developed to quantify geometric distortion via deformable image registration. The MR image is deformed to the CT, generating a deformation field, which is sampled, quantifying geometric distortion as a function of distance from scanner isocenter. Configurability of the QA tool was tested, and results compared to those provided from commercial solutions. Registration accuracy was investigated by repeating the deformable registration step on the initial deformed MR image to define regions with residual distortions. The geometric distortion of four clinical systems was quantified using the customisable QA method presented. Maximum measured distortions varied from 2.2 to 19.4 mm (image parameter and sampling volume dependent). The workflow was successfully customized for different phantom configurations and volunteer imaging studies. Comparison to a vendor supplied solution showed good agreement in regions where the two procedures were sampling the same imaging volume. On a large field of view phantom across various scanners, the QA tool accurately quantified geometric distortions within 17–22 cm from scanner isocenter. Beyond these regions, the geometric integrity of images in clinical applications should be considered with a higher degree of uncertainty due to increased gradient nonlinearity and B_0_ inhomogeneity. This tool has been successfully integrated into routine QA of the MRI scanner utilized for radiotherapy within our department. It enables any low susceptibility MR‐CT compatible phantom to quantify the geometric distortion on any MRI scanner with a configurable, user friendly interface for ease of use and consistency in data collection and analysis.

## 1 INTRODUCTION

Quality assurance (QA) plays a crucial role in ensuring the safety and effectiveness of all equipment used for radiotherapy.^1^, ^2^, ^3^, ^4^, ^5^ Utilization of magnetic resonance imaging (MRI) within radiotherapy departments is increasing for the purposes of tumor delineation,^6^, ^7^, ^8^ response assessment and image guidance.^9^, ^10^, ^11^ Integration of QA programs for these systems is therefore important, in the same way that it is required for CT scanners used for CT‐based radiotherapy simulation.^12^

One component of MRI QA is the quantification of systematic geometric distortions. These distortions arise primarily from inhomogeneities in the main magnetic field (B_0_) and nonlinearities in the magnetic gradient fields. Quantification of these distortions has been widely investigated in the literature.^13^, ^14^, ^15^, ^16^, ^17^, ^18^ While vendor‐supplied correction algorithms aim to reduce these distortions, they may not completely remove them.^17^, ^19^, ^20^ Understanding that these residual distortions are present in images acquired for radiotherapy is important to minimize their effect and ensure the accuracy of tumor and normal tissue delineation for radiotherapy planning. The specific distortion distribution of each system should be quantified, and the geometric integrity of the system verified over time.

This work presents a procedure for vendor neutral distortion assessment useful for implementation of MRI distortion QA for radiotherapy departments. It allows for the distortion quantification over the volume of the object within the imaging field of view (FOV) for a range of different CT and MRI compatible phantoms (regardless of whether commercial or locally constructed). Locally, this procedure has been developed into a user‐friendly graphical user interface (GUI) allowing ease of use with the flexibility to analyze the results and investigate any deviations to the baseline performance and integrated into the MRI routine QA.

## 2 MATERIALS AND METHODS

**FIGURE 1:**
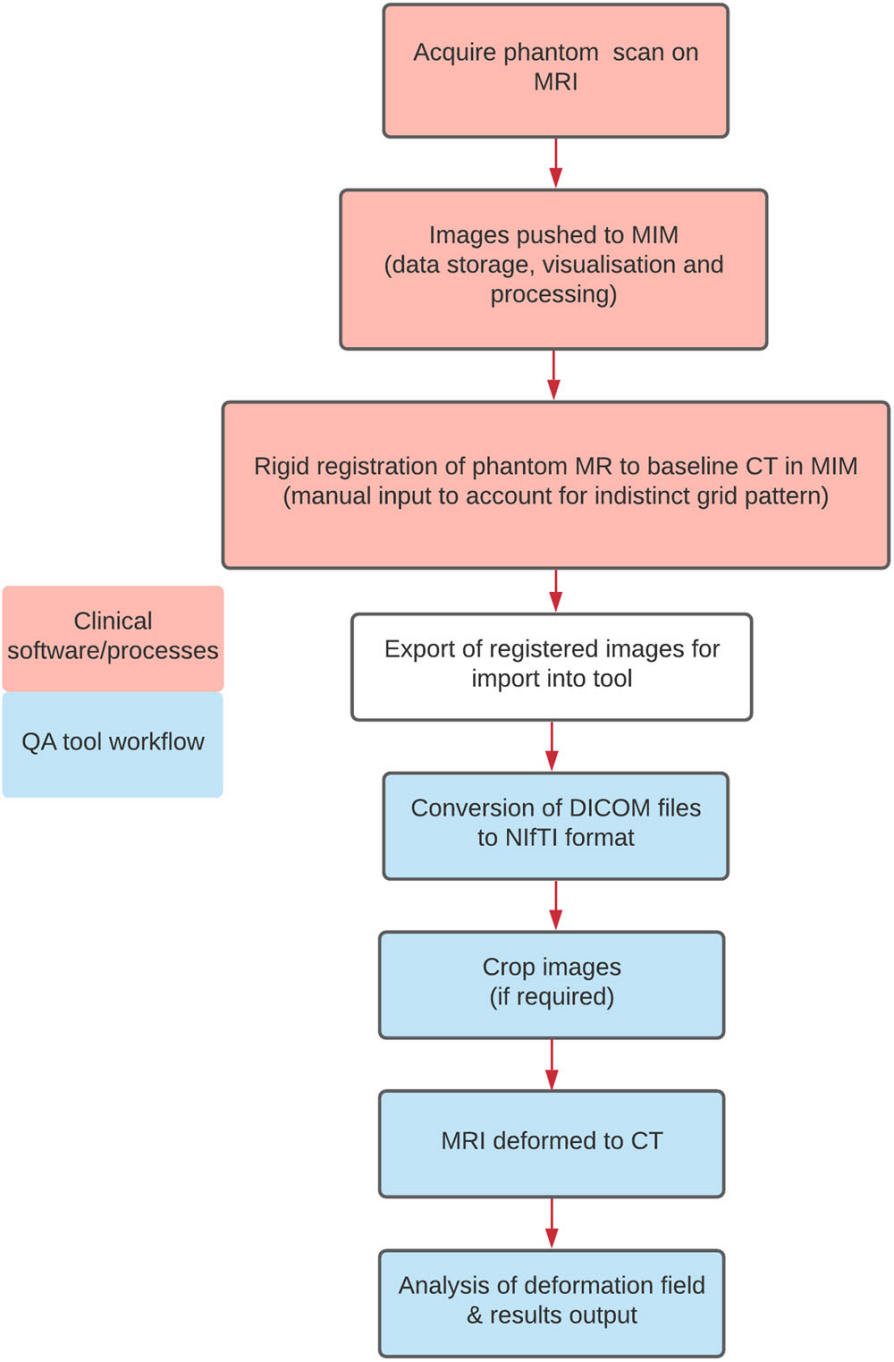
MRI distortion quality assurance (QA) workflow. The blue (bottom) sections represent the steps of the developed QA tool

**FIGURE 2:**
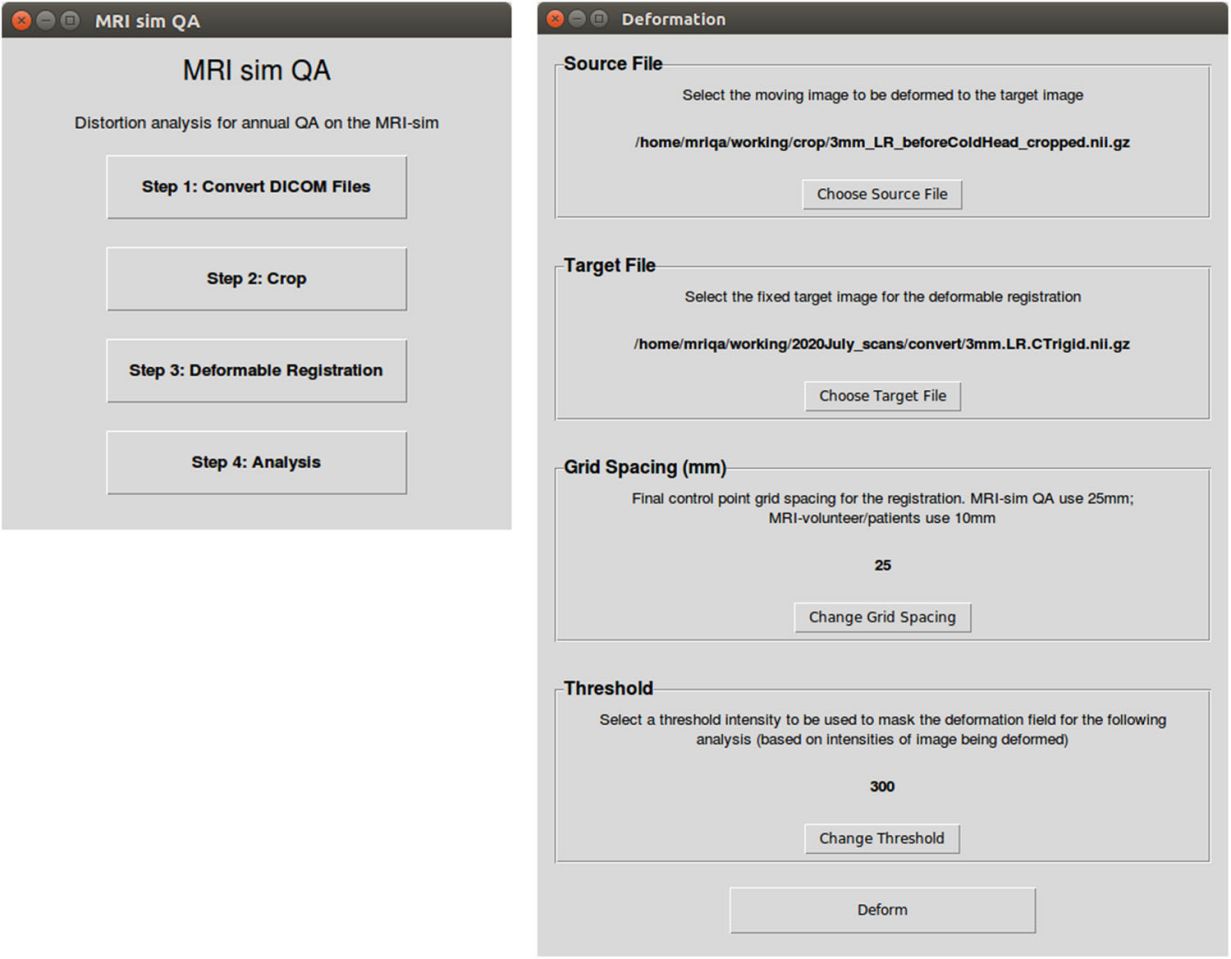
Sample screenshots of the quality assurance (QA) tool. The workflow overview interface on the left, the deformable registration (Step 3) interface on the right

A protocol was developed within the department describing the workflow for routine MRI distortion QA. Initial steps of the workflow are completed using existing clinical software and processes, and for the remaining steps, an in‐house software tool was developed (Figure 1). The QA tool was implemented in‐house using Python 3 and runs on the Ubuntu (Linux) operating system. The tool presents a GUI to the user where they follow each step for data processing, conversion of DICOM data, cropping and deformation of MR datasets to the known geometry (e.g., CT dataset) and analysis of the deformation (and hence distortion) magnitude. The tool uses the open‐source SimpleITK library for image analysis operations and NiftyReg for registration functionality. Some example screenshots of the QA tool GUI are provided in Figure 2.

Other workflow processes such as image visualization and rigid registration (for indistinct regular grid point structure alignment) are performed using commercial software solutions such as treatment planning systems or contouring software. In our department, MIM Maestro software (Medical Image Merge, MIM Software Inc., Cleveland, OH) is used to complete those steps.

### 2.1 Distortion QA procedure

The quantification of geometric distortion implemented in this methodology is based on the registration of two image datasets: MRI deformed to the corresponding CT. This procedure was developed for the use of a large 3D distortion phantom^13^ with isolated grid point markers distinguishable on both CT and MRI. The baseline CT geometry was validated against the known phantom design dimensions and this baseline image can then be used as a reference for future measurements to ensure no variation in the phantom structure. The phantom utilized also has external markers for alignment of the phantom within the scanner (i.e., to an external laser system on each scanner) to maintain setup reproducibility as required for the purposes of routine QA. The QA tool has been developed and optimized around this phantom geometry; however it can be used with any low susceptibility MR/CT compatible phantom with known internal marker geometry discernible on both modalities. Phantoms should be designed to reduce the magnetic susceptibility artefact, that is, by choice in material (e.g., acrylic with similar magnetic susceptibility to water) and/or design shape (e.g., long cylindrical markers).^21^, ^22^ Note that the phantom geometry should always be validated against any manufacturing specifications and assessed for potential variations over time. Additionally, the phantom should be consistently placed in the same location within the scanner for all QA scans (i.e., with the aid of an external laser system with appropriate QA to phantom reference points) to ensure consistency in testing location and conditions.

Images of the phantom were acquired on both imaging modalities and exported to MIM as per departmental procedure for image storage, handling, and transfer. Rigid registration was then performed in MIM resulting in both datasets occupying the same space and resolution. Automation of the rigid registration process proved difficult given the regular, indistinct internal grid geometry of this phantom which could lead to misalignment of the datasets. Rigid registration was hence performed by manually focusing on the center of the phantom, corresponding to the center of the MRI bore, where distortions are known to be at a minimum. Rigid registration must be assessed prior to proceeding with the following steps since a misregistration will lead to an incorrect quantification of system distortion.

The images were then exported for input into the QA tool. The results of each step within the workflow automatically populated the input data requirements for the next step. Alternatively, the user could add input data manually. The first step of the program converted imported DICOM datasets to a NIfTI file format to allow for three‐dimensional data processing and manipulation, with an option to crop the images if necessary (e.g.: to remove any artefacts, ensure images are both the same size). The MRI dataset was then deformably registered to the CT with a B‐spline deformation algorithm in an open‐source registration package (NiftyReg version 1.3.9),^23^ creating a deformation field between the CT and MRI. The B‐spline deformation algorithm overlays a control point grid mesh over the images to be registered, which governs local nonrigid deformations between the two images. The degree to which the image may deform was governed by the resolution of the control points and resulted in a smooth deformation field (assessed by visual assessment of the 3D deformation field). A binary mask is applied to the deformation field on the intensity of the MR image, extracting the deformation at each phantom marker position. The analysis step reads the masked deformation field, and the central position of each marker is extracted to report and visualize the magnitude of distortion between the CT and MRI datasets. Qualitative and quantitative analysis can then be performed on the magnitude of the geometric distortions relative to the position from the MRI isocenter.

Local validation of the methodology and subsequent QA tool was performed with the aforementioned large 3D MRI distortion phantom (physical dimensions: 500 mm diameter, 375 mm height, and 513 mm length: scan length restricted to 370 mm). The phantom was scanned on a 3 T Siemens MAGNETOM Skyra MRI scanner with scan parameters outlined in Table 1 with the images then passed through the distortion assessment procedure outlined. One of the products of this process is an MR image (NIfTI file format), which is deformed to the original CT image (85 cm Brilliance Big Bore CT scanner (Philips Healthcare), FOV 500 mm × 500 mm × 369 mm, voxel size 1 × 1 × 3 mm^3^). Qualitative assessment of the resulting deformed MR image was performed by overlaying the image on the original CT dataset for a visual assessment of the match of the two images.^24^ A second deformable registration of the deformed MR image allows for a quantitative determination of the regions of inaccuracy.

**TABLE 1:** MRI scanners and imaging parameters utilized in this study

Scanner	Sequence	FOV (mm)	Voxel size (mm)	TE (ms)	TR (ms)	Bandwidth (Hz/pixel)	Distortion correction applied	Phantom
Tool development								
3 T Siemens Skyra	2D spin echo	500 × 500 × 369	1.56 × 1.56 × 3	12	2760	445	2D	Large 3D distortion phantom^13^
External utilization and validation								
3 T Siemens Vida	2D spin echo	500 × 500 × 254	1.56 × 1.56 × 2	12	2760	446	2D	Large 3D distortion phantom^13^
3 T GE Discovery MR750w	3D gradient recalled echo	500 × 500 × 369	0.977 × 0.977 × 3	3.1	7.6	112	With and without 3D	Large 3D distortion phantom^13^ and CIRS LF 604‐GS
Phantom customization								
1.5 T Philips Ingenia	3D gradient recalled echo	500 × 500 × 296	0.75 × 0.75 × 2	4.6	13	228	2D	MAGPHAN RT

Abbreviations: FOV, Field of view; TE, Echo Time; TR, Repetition time.

### 2.2 External utilization and validation

Geometric distortion was quantified on the MRI scanners of two external departments using the 3D distortion phantom and the analysis workflow described in Section 21. The sequences and parameters here were selected by each individual department in line with their own scanners and QA requirements and covered both 1.5 T and 3 T systems. Analysis of each department dataset was then to set a relative QA baseline for that scanner, rather than providing distortion quantification between systems. MR images of the phantom were acquired on a 3 T Siemens MAGNETOM Vida and a 3 T GE Discovery MR750w with scan parameters outlined in Table 1.

A CIRS LF 604‐GS phantom^25^ (physical dimensions 330 mm × 276 mm × 300 mm) was also scanned on the 3 T GE Discovery scanner with the 3D GRE sequences and analyzed with the corresponding distortion check analysis software. Results from the commercial and presented methods were compared as a means of validating the locally developed (vendor neutral) method.

### 2.3 Customization

**FIGURE 3:**
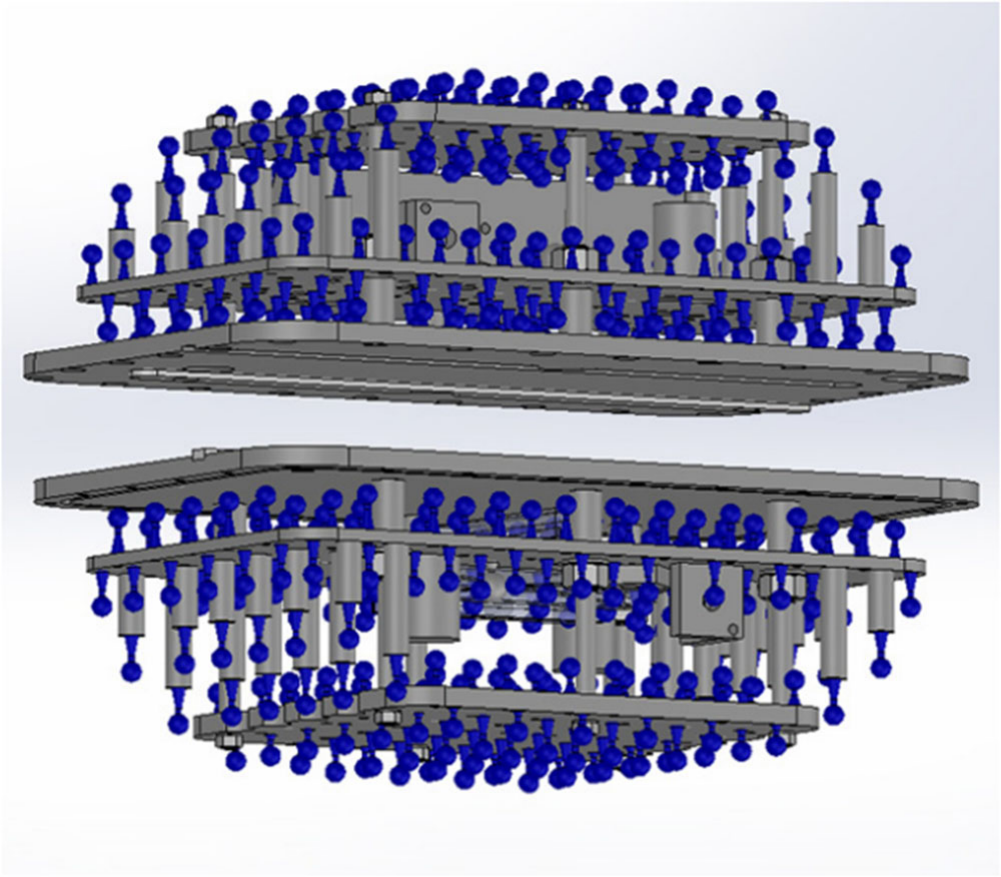
Internal structure of the commercial phantom used to verify the distortion. Blue structures are used in the vendor analysis, blue and grey regions (online version only) are used for the presented methodology

This procedure can be customized for various purposes as may be required for determining distortion or deformation between two images. Two examples tested locally are highlighted in the following sections. MRI and CT datasets of a MAGPHAN RT phantom (The Phantom Laboratory) (physical dimensions 350 mm × 270 mm × 210 mm) were acquired. MR images were acquired on a 1.5 T Philips Ingenia (scan parameters in Table 1). A CT of the phantom was acquired at the same time (85 cm Brilliance Big Bore CT scanner [Philips Healthcare], FOV 500 mm × 500 mm × 296 mm, voxel size 0.98 × 0.98 × 2 mm^3^). An additional masking step of the CT was required to exclude the phantom outer housing (visible on CT but not MRI) to avoid false deformable registration between the two datasets. Comparison of results was made between the distortion results supplied by the phantom company and the presented method. The commercial phantom analysis provided is an on‐line server based licensing solution (https://www.phantomlab.com/smari‐image‐analysis) and is based on the known physical position of the centroid position of each internal spherical fiducial (blue markers shown in Figure 3).^26^ Comparatively, the methodology presented in this work gives distortion information across each voxel within the fluid filled phantom (blue and grey connected structures in Figure 3), since all these regions contain MR signal and are therefore not differentiated by binary masking as with the previous method. Hence, some variation in results is expected since the two methodologies are analyzing the distortion over slightly different volumes.

## 3 RESULTS

### 3.1 Procedure validation

**FIGURE 4:**
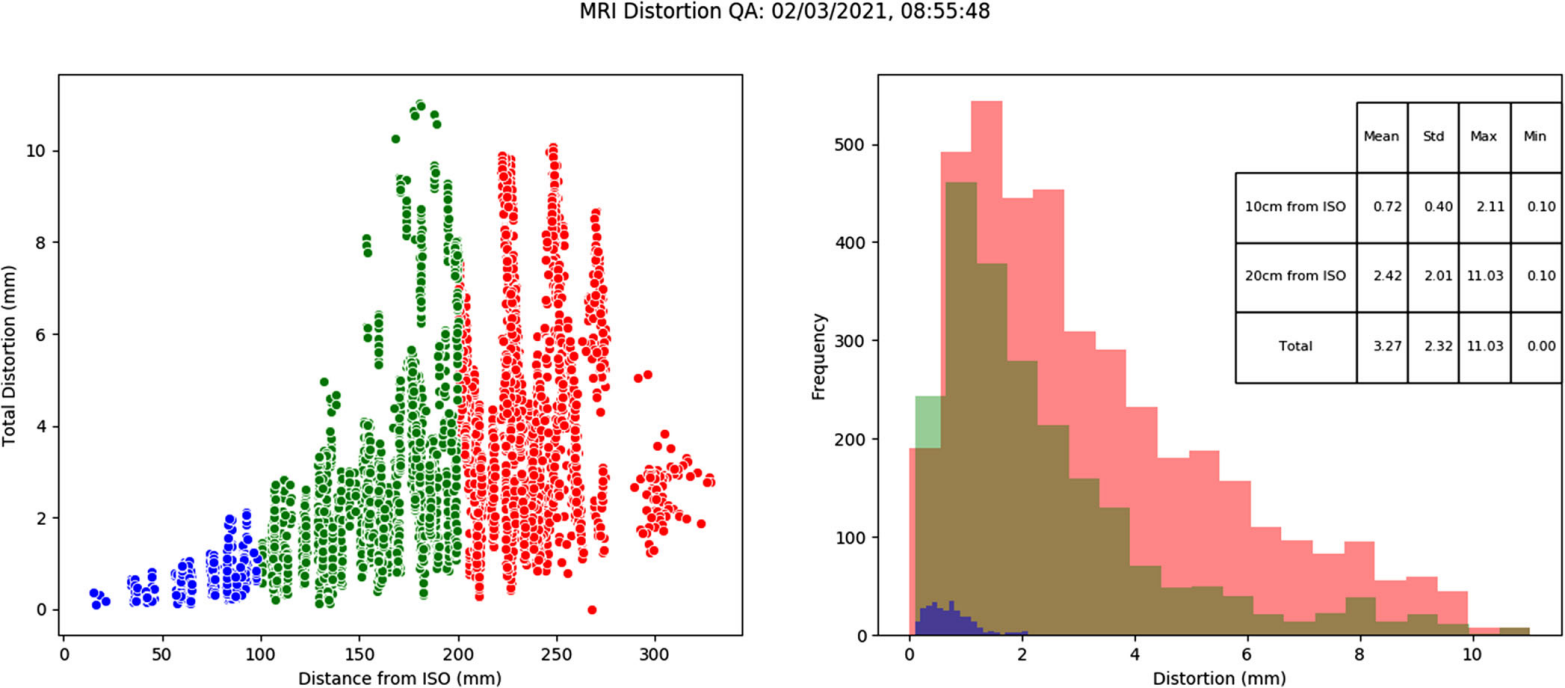
2D representation of the distortion magnitude (left) and frequency of distortion magnitude measurements (right) as a function of 3D distance from isocenter on the 3 T Siemens Skyra for a 2D SE sequence with 2D distortion corrections applied (distance from isocenter: blue: 0–100 mm, green: 100–200 mm and red: > 200 mm; online version only)

Figure 4 shows the graphical results display generated by the QA tool. Two graphs and a table are generated to present the distortion quantification results. The first figure shows the magnitude of distortion of the centroid of each capsule as a function of distance from isocenter, color coded to identify regions 0–100 mm (blue), 100–200 mm (green), and > 200 mm (red) from the isocenter. (Note: the distinct peaks in distortion as a function of distance are a result of reporting discrete radial points from the phantom geometry). The histogram shows the frequency of distortion magnitude, again color coded into the distance from isocenter regions. The purpose of these figures is to enable a quick visual qualitative assessment of any significant variations from the baseline data. This is then supplemented with the quantitative values of distortion within the predefined regions from isocenter. Both the figure and quantitative text file are saved to a specified file location in the analysis process.

**FIGURE 5:**
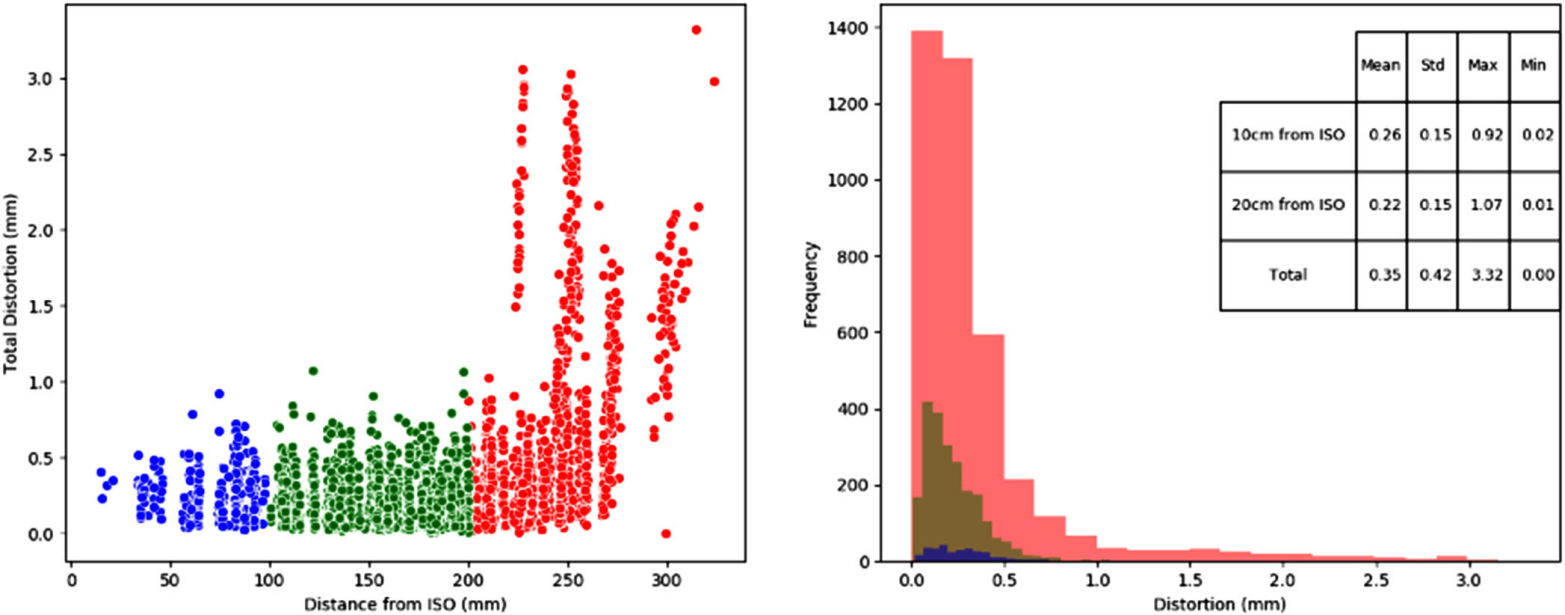
Distribution of regions where the initial image registration was unable to accurately quantify the image distortion in the large field of view (FOV) phantom image (FOV 500 mm × 375 mm × 370 mm) due to large image distortion in peripheral regions of the image

To determine how accurately the QA tool could quantify the magnitude of distortion, the deformed MR image generated in the first registration was registered to the CT for a second time. This enabled the determination of limiting regions in which the QA tool was unable to determine the full magnitude of distortions present for the image dataset provided. For the Siemens 3 T Skyra, the distortions were well detected within a radial distance of 22 cm from the scanner isocenter (Figure 5). It was noted that the areas in which the initial deformable registration was unable to accurately quantify the distortion in some peripheral (superior and inferior) regions of the large FOV image, were where distortions are known to be larger in magnitude.^13^ This additional step provides users with regions where the geometric integrity should be considered with a higher degree of uncertainty in all images acquired for radiotherapy, even in situations where deformable registration may have been performed. This is particularly important for radiotherapy where target volumes may be in these regions.

The imaging protocol utilized for this process was saved on the local MRI scanner for ongoing QA consistency and the entire process successfully integrated into the routine clinical QA. It has been shown to provide reproducible results on analysis on the same datasets and a good constancy QA tool for images acquired at different time points.

### 3.2 External utilization and validation

**FIGURE 6:**
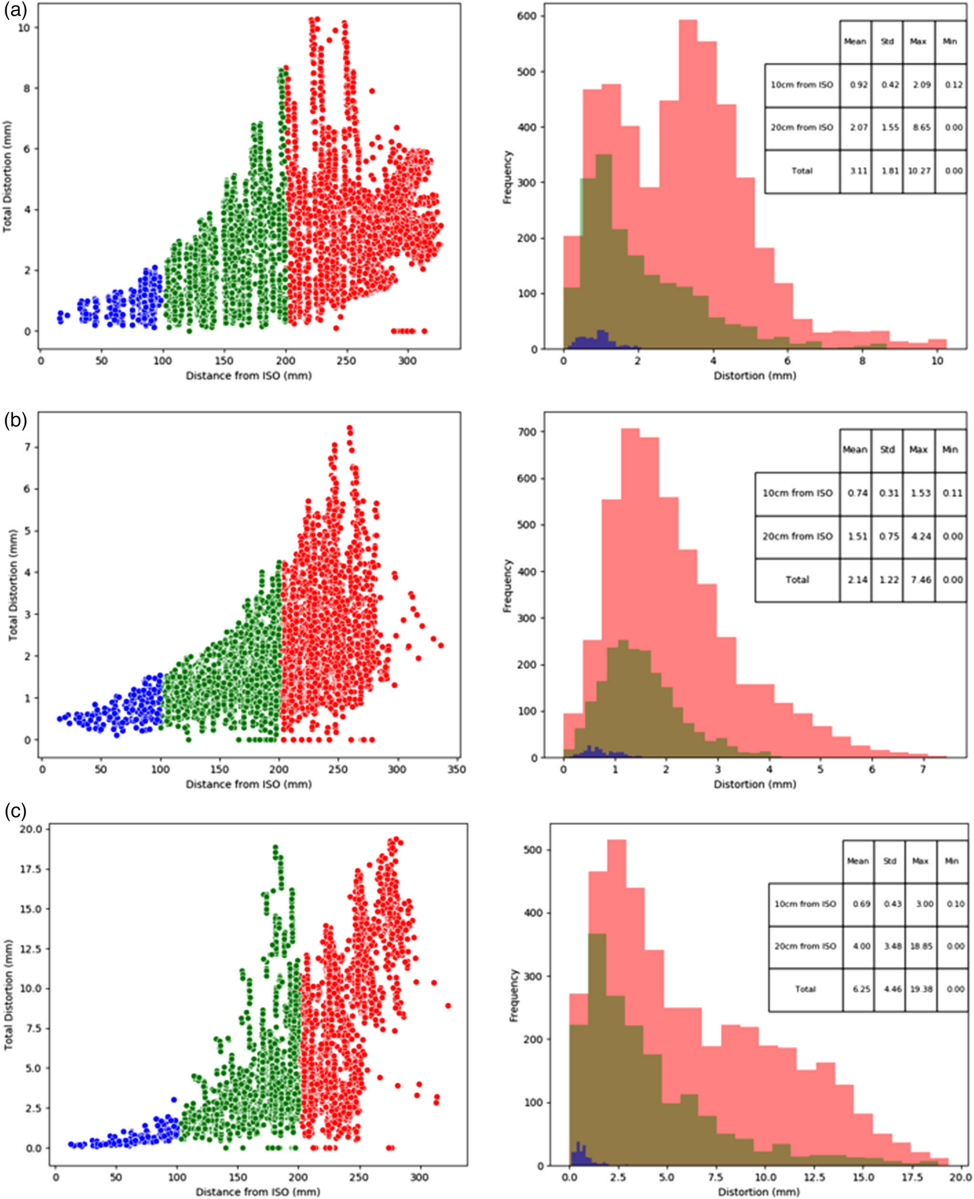
Results from the (a) 3 T MAGNETOM Vida scanner (2D distortion correction applied), and the 3 T GE Discovery scanner with (b) and without (c) 3D distortion correction applied (Note the change in vertical scale). Results show the tools ability to quantify distortion on different scanners and are not meant as a direct comparison of scanner performance given known variations in scan parameters

Figure 6 shows the distortion QA results obtained with the presented workflow on MRI scanners at the two external departments with their specific QA sequences. Given the nature of the variation in sequences and parameters, these results show the feasibility of the software to quantify distortion on different systems and are not meant to serve as a comparison between vendors. Assessment of the residual registration error was also performed on these datasets. It was found that the residual distortions were well detected within a 17‐cm radial distance from the scanner isocenter for all of these images (all with varying imaging parameters).

**FIGURE 7:**
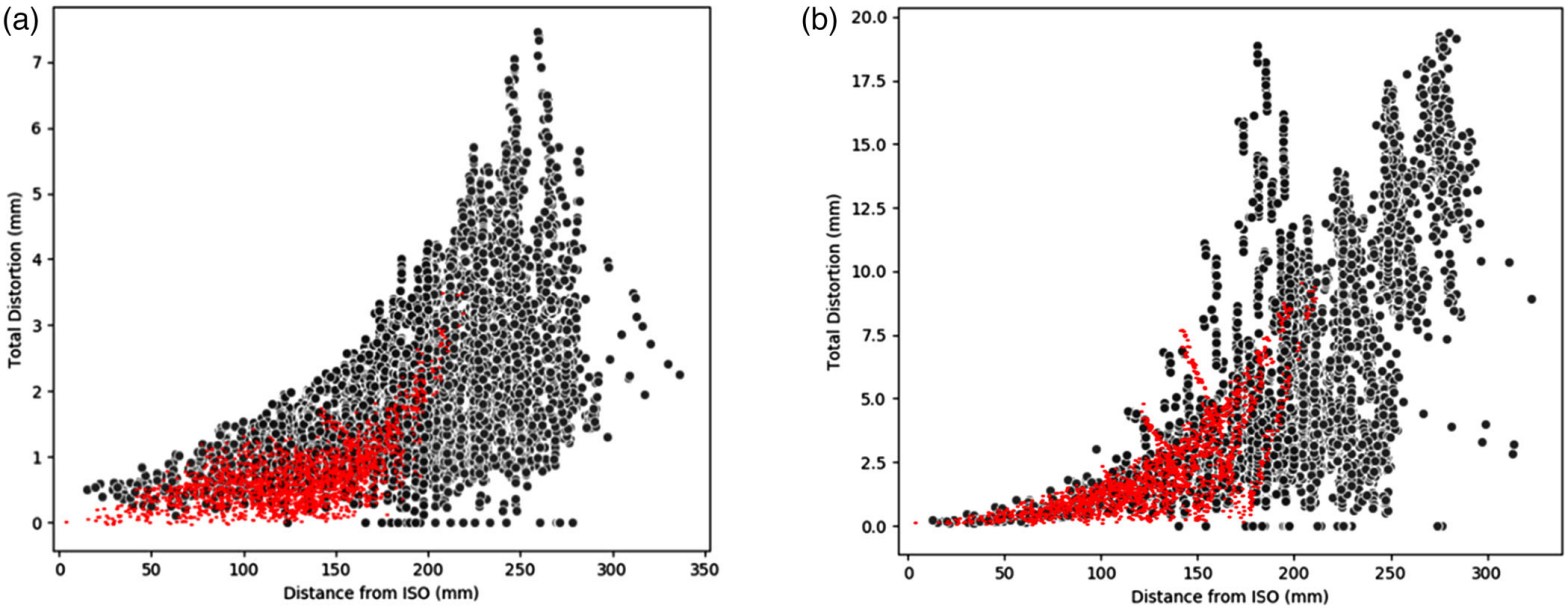
Comparison of results from the presented methodology with a large field of view (FOV) phantom (black) and the commercial CIRS LF 604‐GS product system (red) on the 3 T GE Discovery scanner with 3D (a) and no (b) distortion corrections applied. (Note the change in vertical scale)

Figure 7 shows the comparison of the distortion analysis of the presented QA tool to the commercial CIRS LF 604‐GS solution. There was good agreement between the trends observed in these curves, particularly within a radial distance of 150 mm from isocenter. Within a 150 mm distance from isocenter for the 3D distortion corrected scans, vendor distortion results were 95% quantile 1.1 mm with a median of 0.6 mm compared to a 95% quantile of 1.8 mm and median of 1.0 mm with the presented analysis. Within a 150‐mm distance from isocenter for the nondistortion corrected scans, vendor distortion results were 95% quantile 3.6 mm with a median of 1.3 mm compared to a 95% quantile of 4.4 mm and median of 1.3 mm with the presented analysis. The deviation in distortions reported with increasing distance from isocenter was expected given the phantom used in the presented methodology samples more of the 3D imaging volume with a higher sampling resolution, moving into regions known to be susceptible to increased geometric uncertainty (size difference between the 2 phantoms was 170 mm diameter × 100 mm height × 69 mm length).

### 3.3 Customization

**FIGURE 8:**
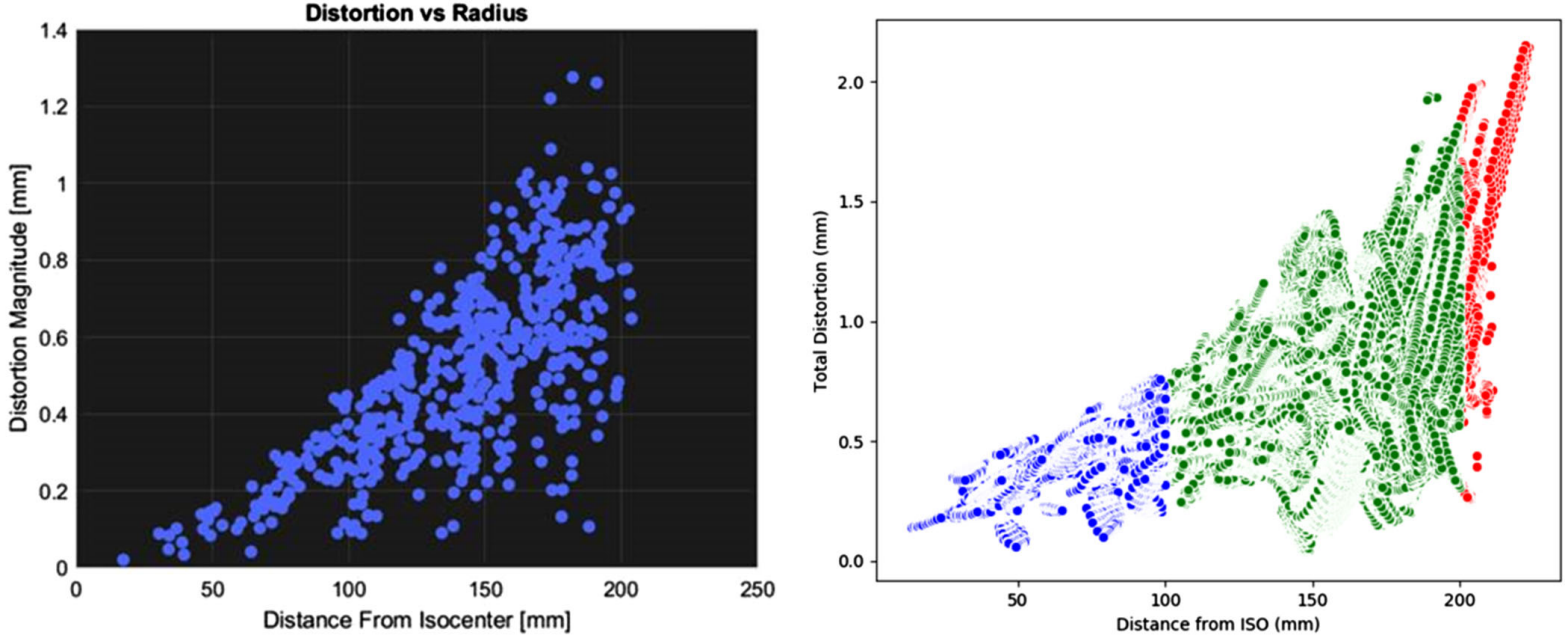
Plots of geometric distortion on the Philips 1.5 T Ingenia as a function of distance from scanner isocenter for the commercial (The Phantom Laboratory) supplied result (left) and the quality assurance (QA) tool workflow (right)

Figure 8 shows the plots of distortion as a function of distance from isocenter for the QA tool and The Phantom Laboratory commercial solution on the same MAGPHAN RT phantom images. The vendor supplied result indicated a maximum distortion of 1.25 mm compared to the developed QA tool, which determined a maximum distortion of 2.15 mm, the latter measuring over a slightly larger volume given the different approaches of the analysis methods. Since the analysis in this customization proof of concept considered all voxels containing the phantoms internal liquid structure, there are significantly more analysis points compared to the vendor results. This analysis could be further customized by the use of image masks to allow sampling over a smaller proportion of data for routine QA purposes. Within a 150 mm distance from isocenter, vendor distortion results showed a 95% quantile of 0.54 mm with a median of 0.24 mm compared to a 95% quantile of 0.94 mm and median of 0.60 mm with the presented analysis.

## 4 DISCUSSION

The methodology and workflow presented in this work provides a way to perform MRI distortion QA on a routine basis, as required for verifying geometric integrity for radiotherapy. It can be utilized during acceptance and commissioning of the scanner, postservice/software upgrades, routine constancy checks of the geometric distortion and for testing new image sequences. It offers a vendor neutral solution in which the methodology can be adapted to meet the specific needs of individual departments and phantoms.

The variation in scanners and sequences presented here in Figure 6 shows the importance of (1) setting up QA baselines and procedures for each scanner and (2) the application of distortion correction algorithms on the scanners. For this investigation, both the Siemens scanners (3 T Skyra and Vida) were tested with a 2D spin echo sequence and the default 2D correction algorithm applied. The distortion measured on these scanners was greater than that measured on the 3 T GE Discovery with the 3D gradient echo sequence applied and the default 3D correction algorithm applied. This is expected given the lack of correction in the third (through‐plane) direction when 2D correction only is applied. The results with no correction applied once again highlight the need to ensure these vendor supplied corrections are applied when acquiring images that are to be used for radiotherapy purposes where the spatial integrity is so critical. While it is acknowledged that a number of other factors also contribute to the distortion (e.g., imaging bandwidth^20^), this study was designed to showcase the feasibility of the proposed methodology, regardless of scan parameters utilized. Hence, it should be noted that the sequence(s) setup for routine QA should be used consistently between the imaging time points, avoiding variations in distortion measurements due to these scan parameters. Users should be mindful of impact potential large FOV limitations may have on QA; however the distribution in these regions should remain consistent in ongoing QA analysis, unless there is a change to the distortion distribution due to systematic sources.

Performing the deformable registration step a second time indicates the limitations of the registration accuracy at the outer regions of large 3D volumes. While there is an underestimation of the total magnitude of the distortion in these regions in the initial workflow, it still provides useful information to the user. This may indicate that the image quality degradation and magnitude of distortion are such that the accuracy needs to be carefully considered for utilization of large FOVs in radiotherapy. It should be noted however that the assessment of distortion in these peripheral regions on clinical images may be more challenging compared to the known geometry phantom‐based method presented here. Any additional geometric corrections applied to anatomy beyond this point may not be a representation of the true anatomy, potentially resulting in dosimetric differences depending on where the treatment beams pass with respect to any distorted anatomical structures. As the distributions of these regions vary between scanners, this should be identified on each system to be utilized for radiotherapy treatment planning.

This methodology is based on the knowledge that image distortions are known to be at a minimum in the center of the MRI bore.^12^ This assumes that there is no distortion to the main B0 field due to differences in susceptibility within the phantom. A limitation of this work is the assumption that there are no susceptibility differences in the phantom being analyzed. It is recommended that a phantom of low magnetic susceptibility be used for such analysis by choice in material (e.g., acrylic with similar magnetic susceptibility to water) and/or design shape (e.g., long cylindrical markers).^21^, ^22^ This methodology is also heavily dependent on the initial rigid registration alignment, and it is imperative that this be performed correctly to ensure what incorrect registration does not result in a systematic shift in the distortion quantification. Particular care must be taken when aligning phantoms of indistinct grid geometries to ensure that corresponding sections of the phantom are aligned in each image.

The configurable nature of this workflow could be extended to separate out the distortion contributions from gradient nonlinearities and B0 field inhomogeneities (the latter assuming use of a phantom of negligible magnetic susceptibility). Two phantom images acquired with frequency encode directions rotated by 180^o^ enable the separation of these two imaging components.^27^, ^28^ The presented workflow could be configured to allow for this analysis to be performed via the method described previously by Walker et al.^13^ For routine QA purposes, this would help in identifying the potential cause of any change in distortion distribution from baseline.

The workflow is configured as an off‐line assessment tool and provides flexibility in its ability to be adapted to a number of different scenarios. The ability of the workflow to be configured makes it potentially suited to a range of different investigational scenarios beyond those presented here. It is based on open‐source software and not for on‐line or clinical applications. The code for the GUI can be made available for use on high powered Linux based environments.

## 5 CONCLUSION

This tool provides a vendor neutral solution for performing MRI distortion annual QA. It enables any MR‐CT compatible phantom (commercial or locally constructed) to quantify the geometric distortion on any MRI scanner utilising deformable image registration methods. Routine distortion QA should be performed with the same imaging sequence(s) to avoid introducing any variations due to the influence of specific scan parameters. This methodology has successfully been integrated into the local departmental QA procedures. The QA tool lends itself to adaptation to be used for any contrast varying phantom visible on CT and MRI. Further developments aim to integrate the automation of the tool further into our existing QA workflow.

## CONFLICT OF INTEREST

The authors declare that there is no conflict of interest that could be perceived as prejudicing the impartiality of the research reported.

## AUTHOR CONTRIBUTIONS

Conceptualization (lead), investigation (lead), methodology (lead), funding acquisition, writing—original draft preparation (lead): Amy Walker. Software (lead), data curation (lead), methodology (supporting), writing—original draft (supporting): Phillip Chlap. Investigation (supporting), visualization (supporting), writing—review and editing (equal): Trent Causer. Investigation (supporting), resources, writing—review and editing (equal): Faisal Mahmood. Investigation (supporting), writing—review and editing (equal): Jarryd Buckley. Conceptualization (supporting), methodology (supporting), writing—original draft (supporting): Lois Holloway.
